# A212 ROLE OF UPADACITINIB AND IUS IN PEDIATRIC PATIENTS WITH REFRACTORY ACUTE SEVERE COLITIS: SINGLE-CENTER CASE SERIES

**DOI:** 10.1093/jcag/gwae059.212

**Published:** 2025-02-10

**Authors:** R Alrwili, K Prowse, M Zachos, R Sharma

**Affiliations:** Gastroenterology, McMaster University, Hamilton, ON, Canada; Gastroenterology, McMaster University, Hamilton, ON, Canada; Gastroenterology, McMaster University, Hamilton, ON, Canada; Pediatric Gastroenterology, McMaster University, Hamilton, ON, Canada

## Abstract

**Background:**

Inflammatory Bowel Disease (IBD) refractory to first-line agents is challenging in pediatrics due to limited therapeutic options, especially in Acute Severe Colitis (ASC). Upadacitinib (UPA) is a selective Janus-Kinase (JAK) inhibitor approved for use in adults. Its use in pediatrics for refractory moderate to severe colitis is off label, but its oral route of administration and quick onset of action make it a promising therapy. Intestinal Ultrasound (IUS) in this context may be a useful tool for monitoring treatment response.

**Aims:**

To describe the efficacy of UPA as a second-line agent for pediatric ASC and report IUS findings to demonstrate its use in monitoring IBD.

**Methods:**

Single-center chart and literature review of pediatric patients with IBD on UPA.

**Results:**

Case 1: A 12-year-old female with pancolonic Ulcerative Colitis (UC) since age 9 maintained on Infliximab (IFX). Despite adequate IFX levels, re-induction and steroids, she had a Pediatric Ulcerative Colitis Activity Index (PUCAI) of 50, elevated C-Reactive Protein (CRP) and fecal calprotectin (FC) leading to repeat admissions. Intravenous (IV) steroids, followed by UPA 45mg for 8 weeks, led to rapid clinical response by Day 4. At discharge, PUCAI was 5 and CRP <5. On Day 20 of UPA, IUS showed normal bowel wall thickness (BWT) and Modified Limberg (ML) score of 0. At 6 months, she remains in steroid-free remission with a normal FC.

Case 2: A 16-year-old female presented with a Pediatric Crohn’s Disease Activity Index (PCDAI) of 45 and was diagnosed with Crohn’s colitis with perianal fistula. Due to inadequate response to 6 days of IV steroids, she received IFX. Despite robust IFX levels, IUS showed pancolonic inflammation with increased BWT of 4.6 mm and ML score of 1-2. [1] She was readmitted with severe anemia and elevated CRP. Concurrent C. jejuni infection was treated without symptom resolution. Given severe colitis despite IFX and steroids, UPA 45mg for 12 weeks was started with clinical response and discharge by Day 4; PCDAI of 20. At 1 month, she remains asymptomatic on a steroid taper with a normal hemoglobin. Repeat IUS and FC is pending.

Case 3: A 15-year-old male with IBD favouring UC since age 14, who initially did well on steroids and IFX was re-admitted with a PUCAI of 65 and elevated FC while weaning steroids. Repeat endoscopy showed Mayo 2 colitis to hepatic flexure correlating to increased BWT of 3.5 mm and ML score of 2 on IUS. Despite appropriate IFX levels, due to steroid dependence at discharge (PUCAI 25), UPA 45mg for 8 weeks was started as an outpatient. By Day 21 his PUCAI was 0, with normal FC. At 2 months, IUS showed normal BWT and ML Score 0 with steroid-free remission.

**Conclusions:**

These cases demonstrate the efficacy of UPA in refractory pediatric ASC with rapid clinical response, normalization of biochemical tests and IUS findings.

**Funding Agencies:**

None

